# Role of Wheat *Phosphorus Starvation Tolerance 1* Genes in Phosphorus Acquisition and Root Architecture

**DOI:** 10.3390/genes13030487

**Published:** 2022-03-10

**Authors:** Hina Abbas, Muhammad Kashif Naeem, Marya Rubab, Emilie Widemann, Muhammad Uzair, Nageen Zahra, Bilal Saleem, Amna Abdul Rahim, Safeena Inam, Muhammad Imran, Farhan Hafeez, Muhammad Ramzan Khan, Sarfraz Shafiq

**Affiliations:** 1 National Agricultural Research Centre, National Institute for Genomics and Advanced Biotechnology, Islamabad 44000, Pakistan; hina87abbas@gmail.com (H.A.); kashifuaar102@hotmail.com (M.K.N.); maryarubab27@gmail.com (M.R.); uzairbreeder@gmail.com (M.U.); nageenzahra@hotmail.com (N.Z.); bilal.saleem@bs.qau.edu.pk (B.S.); amnaraheem786@gmail.com (A.A.R.); safeena.inam@gmail.com (S.I.); 2 Department of Biotechnology, University of Kotli Azad Jammu and Kashmir, Islamabad 11100, Pakistan; 3 Institut de Biologie Moléculaire des Plantes, CNRS-Université de Strasbourg, 67084 Strasbourg, France; ewidema4@uwo.ca; 4 State Key Laboratory for Conservation and Utilization of Subtropical Agro-Bioresources, College of Agriculture, South China Agriculture University, Guangzhou 510642, China; imran_m1303@yahoo.com; 5 Department of Environmental Sciences, Abbottabad Campus, COMSATS University Islamabad, University Road, Abbottabad 22060, Pakistan; drfarhan@cuiatd.edu.pk; 6 Department of Anatomy and Cell Biology, University of Western Ontario, 1151 Richmond Str., London, ON N6A5B8, Canada

**Keywords:** *PSTOL1*, phosphorus, root phenotyping, genome-wide analysis, wheat

## Abstract

The wheat plant requires elevated phosphorus levels for its normal growth and yield, but continuously depleting non-renewable phosphorus reserves in the soil is one of the biggest challenges in agricultural production worldwide. The *Phosphorus Starvation Tolerance 1* (*PSTOL1*) gene has been reported to play a key role in efficient P uptake, deeper rooting, and high yield in rice. However, the function of the *PSTOL1* gene in wheat is still unclear. In this study, a total of 22 *PSTOL1* orthologs were identified in the wheat genome, and found that wheat *PSTOL1* orthologs are unevenly distributed on chromosomes, and these genes were under strong purifying selection. Under different phosphorus regimes, wheat *PSTOL1* genes showed differential expression patterns in different tissues. These results strengthen the classification of Pakistan-13 as a P-efficient cultivar and Shafaq-06 as a P-inefficient cultivar. Phenotypic characterization demonstrated that Pakistan-13 wheat cultivar has significantly increased P uptake, root length, root volume, and root surface area compared to Shafaq-06. Some wheat *PSTOL1* orthologs are co-localized with phosphorus starvation’s related quantitative trait loci (QTLs), suggesting their potential role in phosphorus use efficiency. Altogether, these results highlight the role of the wheat *PSTOL1* genes in wheat P uptake, root architecture, and efficient plant growth. This comprehensive study will be helpful for devising sustainable strategies for wheat crop production and adaptation to phosphorus insufficiency.

## 1. Introduction

Phosphorus is one of the primary and vital minerals a plant needs, especially for plant growth. It represents about 0.2% of the dry weight of plants and is a crucial part of the energy unit ATP, phospholipids, and nucleic acids [1]. Phosphorus is present abundantly in tropical soil, yet its bioavailability is relatively low. Plants can uptake phosphorus in the form of orthophosphate (P_i_), but most of the phosphorus is either present in the form of organic compounds or fixed in the soil clay by iron oxides and aluminum [1]. Phosphorus is essential for seed germination, tillering, and seed development in wheat. Compared to other food crops, wheat (*Triticum aestivum* L.) requires elevated phosphorus (P) levels. According to an estimate, wheat removes about 2.5–8 kg of phosphate per ton of grain from the soil [2]. Soil with lower phosphorus content has a constraint for biomass and grain yield and greatly hinders the growth and development of wheat. To increase the bioavailability of phosphorus and overcome its deficit in the soil, there is a need to develop crops having deeper rooting systems, nutrient efficiency, and higher yield under phosphorus starvation conditions. This also emphasizes the development of wheat varieties that will perform better under phosphorus-deficient conditions and ultimately improve wheat productivity and food security [3].

In response to phosphorus starvation, the plant exhibits several morphological changes, including extended root, increased dry root weight and root surface area, and decreased root diameter [4]. Some plants secrete mucilage upon phosphorus deficiency, while growing conditions also affect the responses against phosphorus deficiency [4]. According to [5], during insufficient phosphorus levels in soil, a decrease in biomass, photosynthesis, and nitrogen fixation ability was observed in mashbean and mungbean plants. Barley plants (*Hordeum vulgare*) showed an increase in root to shoot ratio, decrease in shoot weight, biomass, and tillering, and a minor reduction in chlorophyll content when grown hydroponically in a phosphorus-deficient environment [6]. In rice (*Oryza sativa*), the phosphorus starvation caused a reduction in nodal root number, plant phosphorus content, dry weight of shoots, plant height, and plant tiller number. These rice plants also exhibited a decline in the density of large lateral roots and in the length of both large and small lateral roots, along with an increase in thickness and length of root hairs [7].

Along with morphological responses, plants trigger many molecular responses to cope with phosphorus deficiency in the soil. Plants reprogram their metabolism to increase phosphorus use efficiency, and they also regulate the production of high-affinity phosphorus transporters to control the inflow of extracellular inorganic phosphate [8]. Many plant factors regulate the *PSR* (*phosphate starvation responsive*) genes, with microRNAs being the new players. The microRNAs regulate Pi homeostasis in plants and signaling during P deficiency [9]. Similar factors were then identified in P-deficiency responsive genes of tomato (*TPSI1*), *Medicago truncatula* (*Mt4*) [10,11], *Arabidopsis thaliana* (*At4* and *AtIPS_1_*) [12,13], and *Oryza sativa* (*OsPI_1_*) [14]. Genes encoding phosphate transporter proteins were found to be involved in coping against phosphorus stress by generating a symbiotic relationship with ectomycorrhiza in Masson pine (*Pinus massoniana*) [15]. Among other P-deficiency tolerance-inducing genes, *PSTOL1* is one of the critical genes. The *Pup1*-specific protein kinase was discovered by [16] and named as *Phosphorus Starvation Tolerance1* (*PSTOL1*) gene in rice (*Oryza sativa*). It was observed that when the *OsPSTOL1* gene was over-expressed in plants and grown under phosphorus-deficient soil, a significant increase in yield and biomass of rice occurred. *PSTOL1* gene acts by increasing the early growth of roots, enabling the plants to acquire more phosphorus from P-deficient soil [16]. The strategy of identifying *PSTOL1* orthologs in different crops and targeting their expression will help to develop crops that proliferate in phosphorus-deficient soil with more yield and biomass. However, the function of *PSTOL1* in wheat is yet to be investigated.

Here, we first identified *PSTOL1* orthologs in the wheat genome and then comprehensively analyzed them using bioinformatics analyses, such as phylogenetic analysis, gene structure, domain organization, chromosomal location, and the cis-regulatory elements in the wheat *PSTOL1s* promoters. Furthermore, the expression of wheat *PSTOL1s,* morpho-physiological traits, and high-throughput root phenotyping was investigated in two different wheat cultivars under different phosphorus regimes. Thus, our comprehensive analysis provides a fundamental understanding of wheat *PSTOL1s* genes in phosphorus uptake and plant growth. Wheat *PSTOL1s* orthologs can be targeted to make wheat cultivars producing a high yield of grains in P-deficient soils. This study also aims to reduce the use of phosphorus fertilizers, preserve the continuously depleting phosphate rock reserves, and build better wheat cultivars capable of growing even under low phosphorus conditions.

## 2. Materials and Methods

### 2.1. Plant Materials and Growing Conditions

Two wheat varieties, Pakistan-13 and Shafaq-06, were selected to understand the phosphorus-deficient response at the morphological level. The seed surface was sterilized with Clorox and thoroughly washed with distilled water. Seeds were incubated on moistened filter paper in Petri plates. After 2–3 days, the seedlings were shifted in the labeled plastic bags. These plastic bags (8 × 4 inches) were filled with a soil mixture of soil: sand at the ratio of 2:1. One seedling was transplanted in each bag, while each genotype had five observations for one treatment. After a fortnight, the phosphorus treatment was initiated. Control plants (C) were given normal tap water (0.02 mM P content), phosphorus-treated plants (+P) were watered with 0.5 mM KH_2_PO_4_ (potassium phosphate) solution, and the plants grown in phosphorus-deficient environments (−P) were irrigated with 0.005 mM KH_2_PO_4_ solution [17]. These treatments were provided for 15 days. After that, morpho-physiological parameters were taken, and RNA sampling was carried-out for further experimentation.

### 2.2. Identification of PSTOL1 Orthologs in Wheat

The *PSTOL1* nucleotide and protein sequences of *Oryza sativa* were retrieved from NCBI (GenBank: BAK26566.1). These were used as a query for BLASTN and BLASTP at EnsemblPlants (https://plants.ensembl.org/index.html) [18]. Duplicate hits were removed, and the cut-off values of identity >50% and *E-value* (1e-5) were used for sequence reliability. Further, to find the evolutionary relationship among the wheat and closely related species, i.e., *Oryza sativa, Brachypodium distachyon, Arabidopsis thaliana, Sorghum bicolor,* and *Zea mays,* *OsPSTOL1* protein and nucleotide sequences (BAK26566.1) were used as queries to BLASTN and BLASTP against these related species. For this purpose, we kept the cut-off value of *E-value* = 1e-5 and identity ≤ 60%.

### 2.3. Gene Structural Analyses

The default modes of the SMART (https://smart.embl.de/smart/set_mode.cgi?NORMAL=1) tool [19] and MEME (https://meme suite.org/meme/tools/meme) Version 5.3.3 [20] were used to predict conserved domains and motifs in the identified 48 *PSTOL1* orthologs in wheat. Sequences were filtered after domain analysis, and 22 sequences were kept that had the conserved Ser/Thr kinase domain (Pfam identifier PF00069.18) of the protein kinase family. GSDS2.0 (http://gsds.gao-lab.org/) online tool was used to draw the gene structure of selected sequences [21]. TBtools was used to illustrate the motif, domain, and gene structure patterns of these 22 genes [22].

### 2.4. Neighbor-Joining Tree Analysis

ClustalW was used for multiple protein sequences alignment of 22 wheat *PSTOL1* orthologs and 41 identified orthologs from other species [23]. The alignment was further used to build a phylogenetic tree using the neighbor-joining (NJ) method with 1000 bootstrap replications in MEGAX software [24]. The phylogenetic tree of 22 *TaPSTOL1* orthologs and the second tree of *TaPSTOL1,* along with 74 identified orthologs from other species, were constructed using MEGAX. Tree roots were illustrated using iTOL v.6 (https://smart.embl.de/smart/set_mode.cgi?NORMAL=1) [25].

### 2.5. Cis-Regulatory Elements Analysis within Promoter Region

*Cis*-regulatory elements were identified in the promoter sequences (about 1500 bp upstream region) of 22 *TaPSTOL1* genes, as previously described in [26]. The online tool PlantCare (http://bioinformatics.psb.ugent.be/webtools/plantcare/html/) [27] was used to identify *cis*-elements in the upstream promoter sequences. The results were visualized as a heatmap in TBtools v.1.082.

### 2.6. Evolutionary Relationship Analyses of TaPSTOL1 with Ancestral Species

Proteomes and GFF3 files of four wheat ancestors (*Triticum urartu, Triticum dicoccoides, Triticum turgidum, Aegilopes tauschii*) were downloaded from the EnsemblPlants database. Similar files for *Triticum aestivum* L. were downloaded from IWGSC (iwgsc_refseqv1.1) wheat database [28]. BLASTP was conducted for all five proteomes on Linux with in-house scripting. BLAST and GFF3 files were further used to predict collinearity through the MCScanX algorithm [29]. Segmental duplicates were further identified from collinearity files, gene pairs with ancestral species and self-syntenic gene pairs of wheat were visualized using advanced circus in TBtools v.1.082.

For performing Ka/Ks analysis, the coding sequences of the identified segmental duplicates were acquired, and multiple sequence alignment was conducted using ClustalW. In-house *Perl* script was built based on ParaAT 2.0 to translate the multiple sequence alignment into axt format [30]. After getting the alignment in the required format, the Ka/Ks (nonsynonymous per synonymous substitution rates) were calculated with Nei and Gojobori method using the KaKs 2.0 calculator [31]. The divergence time between duplicated genes was calculated by T = Ks/2r [32] (Supplementary Table S1).

### 2.7. Transcriptome Data Analysis

RNA-seq data were retrieved from wheat expression browser expVIP (http://www.wheat-expression.com/) [33,34]. Expression data of 22 orthologs in TPM (transcript per million) value were collected from the root and shoot tissues at the vegetative stage in both control and phosphorus-deficient treatments. Data on developing grain, seed coat, spike, rachis, and ovary were also taken. TPM values were used to generate a heatmap in TBtools. The upregulated and downregulated genes were further validated among two wheat cultivars through expression profiling. Expression primers of selected genes were designed using Primer-BLAST of NCBI [35], primers information was enlisted (Supplementary Table S2).

### 2.8. RNA Purification, cDNA Synthesis and Quantitative Real-Time PCR (qRT-PCR)

Plants were harvested for RNA sampling after 15 days of treatment. Two biological replicates from each treatment of both cultivars were retained for RNA sampling. Primary roots of each plant were cut with sterile scissors and cleaned with 70% ethanol. Samples were enveloped in aluminum foil and immediately placed in liquid nitrogen. RNA extraction from the root samples (2 g) was performed using the TRIzol^®^ method according to [36] with the Na-precipitation method. Both biological replicates were pooled after RNA extraction. After normalization, complementary DNA synthesis was performed using a Thermo Scientific Revert Aid-Reverse Transcriptase kit (K1691), according to the manufacturer’s protocol. Further, real-time quantitative PCR (Applied Biosystems^®^ 7900 HT Fast RT-PCR) with StepOnePlus software was used to check the expression of the selected orthologs among three P treatment growth conditions (C, −P, and +P). SYBER-Green kit (Taq Man) was used for making reaction mixture. Both *Actin* and *Tubulin* reference genes were initially used, and both demonstrated a similar expression pattern. Hence, for further RT-PCR experiment reference gene *TaActin* was used to normalize the relative expression data. Relative expression values were evaluated by using the 2ΔΔCT method [37].

### 2.9. Co-Localization of TaPSTOL1 Orthologs with Phosphorus Starvation-Related QTLs

To identify the localization of *T. aestivum PSTOL1* orthologs with QTLs for morpho-physiological and biochemical traits under phosphorus starvation, QTLs and linked molecular markers were retrieved from previous publications [38,39,40,41,42,43], and co-localization was shown as described previously in [44]. To obtain the physical position, each marker sequence or marker name was BLAST against the grain gene database [45]. *TaPSTOL1* orthologs were co-localized with phosphorus stress-related QTLs and were displayed by using MapChart software [46]. QTLs co-localized with the *TaPSTOL1* orthologs were visualized by red color.

### 2.10. Morphological Evaluation

After 15 days of treatment, the shoot and root of each treatment were harvested. Morpho-physiological traits, i.e., tiller per plant, shoot length (cm), chlorophyll content (ug/g), shoot and root phosphorus content (mg/kg), and high-throughput root phenotyping were recorded from three biological replicates.

### 2.11. Chlorophyll Content Measurement

Three biological and three technical replicates from the first leaves of each cultivar were taken for chlorophyll contents estimation. Chlorophyll contents were measured by chemically extracting the chlorophyll from leaves using the proposed method [47]. The chl a and chl b contents were then calculated according to [48].

### 2.12. Phosphorus Content Estimation

Root and shoot phosphorus contents were measured for each treatment. About 0.25 g tissue sample was taken for digestion. The digested samples were then analyzed to quantify phosphorus content using an atomic absorption spectrometer [49]. Raw data were used to calculate the P concentration expressed in mg/kg dry weight.

### 2.13. High-Throughput Root Phenotyping

The washed and cleaned roots were placed in a root scanning glass tray (8 × 12 inches). The tray was filled with water to spread the roots evenly. Roots were scanned using hp^®^ Scanjet 5590 at a gray-white scale and 300 dpi resolution. The images were used to find root traits using RhizoVision Explorer-interactive software for generalized root image analysis [50]. Root traits such as total root length, root volume, and root surface area were estimated.

### 2.14. Statistical Analysis

Bar charts were made in Origin8.5 [51] for all morphological and physiochemical parameters to demonstrate the descriptive statistics. *t*-test was employed to understand the genetic variations among these studied traits of both genotypes under phosphorus deficient (−P), control (C), and sufficient phosphorus (+P) conditions.

## 3. Results

### 3.1. Wheat PSTOL1 Sequence Identification, Phylogenetic, Gene Structure, Motif, and Domain Analyses

A sequence similarity search through BLASTN and BLASTP was performed using the query sequence of *OsPSTOL1*, and a large number (approximately 103) of *PSTOL1* homologs in wheat were identified. Based on the set threshold (>50% and 1e-5), 48 members were filtered out. The BLAST search found orthologs with high similarity on chromosomes 3B, 6A, 6D, and 3A with 92%, 90%, 90%, and 89% identity, respectively. These orthologs were widely spread over 1A, 1B, 2B, 2D, 3A, 3B, 3D, 5B, 5D, 6A, and 6D chromosomes (Supplementary Figures S1). These *TaPSTOL1* orthologs were also analyzed using ExPASy, and we identified gene lengths ranging from 1146 to 2603 bp, which encode proteins comprising 381–720 amino acids, with predicted molecular weight ranging from 43,148.18 to 79,132.44 Da (Supplementary Table S3).

**Figure 1:**
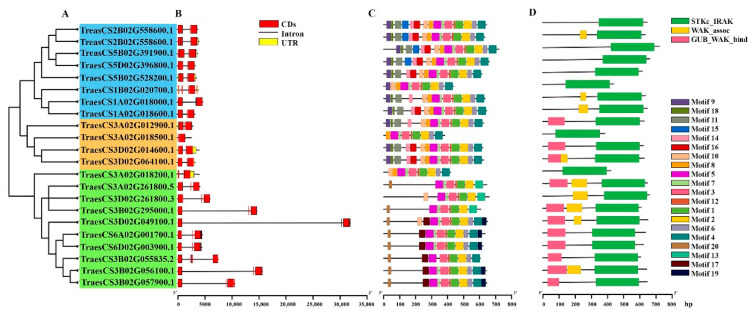
Phylogenetic tree, gene structure, motif, and domain analyses. (**A**) Phylogenetic tree constructed on MEGAX using NJ method (1000 replications) representing three groups I (blue), II (orange), and III (green). (**B**) Exon, intron, and UTRs analyses performed on GSDS 2.0. (**C**) Conserved motifs identified with MEME tool. Each motif is shown with different color, as demonstrated in the legends. (**D**) Conserved domains identified using SMART tool. Green rectangle represents the S_TKc domain, pink shows the GUB_WAK_bind domain, and yellow indicates the WAK_association domain.

A phylogenetic tree of 22 wheat orthologs was constructed using MEGAX to understand the evolutionary relationship between *TaPSTOL1* genes and determine the structural diversity (Figure 1A). The phylogenetic tree could be divided into three subgroups based on topology and alignment. Group I (highlighted in blue) had eight orthologs belonging to chromosomes 2B, 5D, 5B, 1B, and 1A. Group II (highlighted in orange) had four orthologs that were present on chromosomes 3A and 3D. Group III (highlighted in green) was the largest clade and had 10 orthologs on 3A, 3D, 3B, 6A, and 6D. To identify the structural diversity of *PSTOL1* wheat orthologs, gene structures were determined. The results showed that *TaPSTOL1* orthologs differ in the number and length of introns/exons. All *TaPSTOL1* orthologs contained exons and introns, with 1 ortholog containing only 1 intron, 13 orthologs containing 2 introns, 7 orthologs had 3 introns, and 1 ortholog had 4 introns. Variations among the intron numbers are probably due to the functional divergence of these orthologs. Additionally, eight orthologs had untranslated regions (UTRs) (Figure 1B).

The 22 wheat *PSTOL1* orthologs were searched against the MEME suite for identifying the conserved motifs. A total of 20 conserved motifs were identified in *TaPSTOL1* orthologs (Figure 1C and Supplementary Table S4). Six identified motifs were part of the functional and conserved S_TKc domain. Motif 20, only predicted in 6 orthologs, was part of the GUB_WAK_bind domain, and motifs 9,11, and 18 were also part of the GUB_WAK_bind domain in 3 orthologs. Motifs 8 and 10 were related to the WAK_association domain in 3 orthologs (Figure 1C). The conserved domain in the *PSTOL1* gene of *Oryza sativa* is S_TKc (Ser/Thr Kinase) (UniProtKB-A0A0H3VD96). SMART database was used to identify domains in the 48 orthologs. A total of 22 of them were found to have S_TKc domain, along with that 11 orthologs also had GUB_WAK_bind domain, which is a cysteine residue-rich wall-associated receptor, galacturonan binding kinase domain. This domain is the extracellular component of the S_TKc domain and binds to the pectin in the cell wall. Moreover, nine orthologs also had another domain, the WAK_association domain on the C terminal of the GUB_WAK_bind domain (Figure 1D).

### 3.2. Evolutionary Relationship of TaPSTOL1 Genes

**Figure 2:**
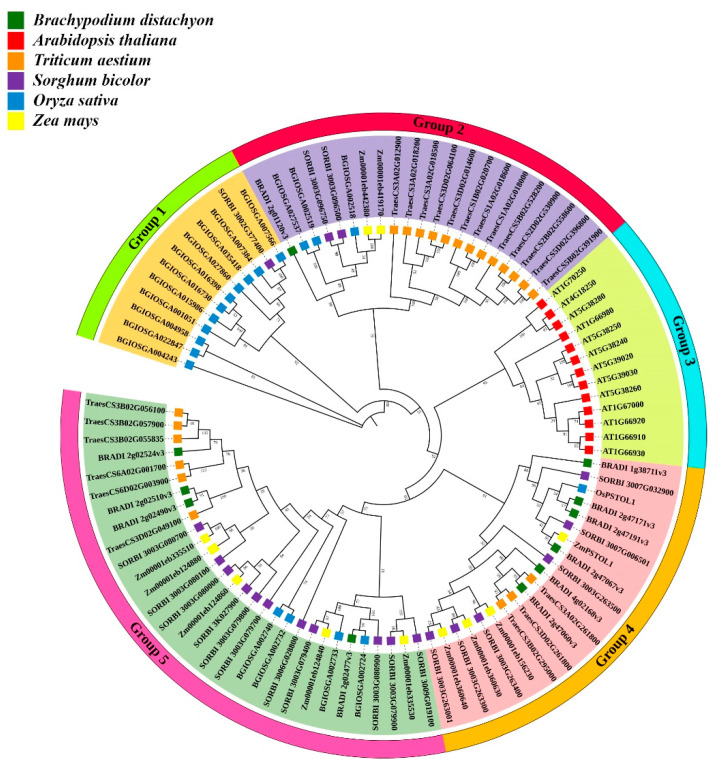
*PSTOL1* wheat orthologs in *Triticum aestivum* L., *Arabidopsis thaliana*, *Sorghum bicolor*, *Zea mays*, and *Oryza sativa*. A total of 96 homologs were aligned in ClustalW, and a phylogenetic tree was constructed in MEGAX using the NJ method. The percentage of replication is presented next to the branches. Homologs from different species are shown with variable-colored squares, such as green color for *Brachypodium distachyon*, red for *Arabidopsis thaliana*, brown for *Triticum aestivum* L., purple for *Sorghum bicolor*, blue for *Oryza sativa,* and yellow for *Zea mays*.

We further investigated the evolutionary relationship of wheat *PSTOL1* orthologs with other closely related species such as *Brachypodium distachyon*, *Arabidopsis thaliana*, *Sorghum bicolor*, *Oryza sativa*, and *Zea mays*. The phylogenetic tree was constructed using the neighbor-joining method with 1000 bootstrap replications on MEGAX (Figure 2). A total of 22 orthologs from *Triticum aestivum* L., 11 orthologs from *B. distachyon*, 13 from *A. thaliana*, 20 from *S. bicolor*, 11 from *Z. mays*, 19 from *O. sativa* were employed to construct the phylogenetic tree. *PSTOL1* genes could be classified into five distinct groups based on topology. Among all 5 groups, out of 22 *TaPSTOL1* orthologs, 13 genes were found in group 2 having a separate clade, while 6 genes were found in group 5, making a close association with *B. distachyon*. Interestingly, three *TaPSTOL1* genes belonging to chromosome 3 (A, B, and D sub-genomes) were found in group 4 along with *OsPSTOL1* and *ZmPSTOL1*. It was also observed from this evolutionary analysis that all the 22 wheat *PSTOL1* orthologs were closely related to monocot species while quite diverse from the dicot species *A. thaliana*. These results suggest a quite distant relationship of *TaPSTOL1* genes with both *O. sativa* and *Z. mays*.

### 3.3. Cis-Regulatory Elements Analysis of Promoter Regions of Wheat PSTOL1 Genes

**Figure 3:**
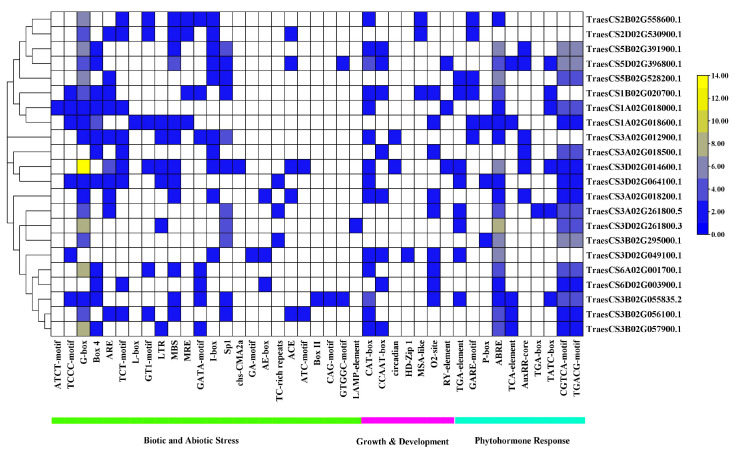
Cis-regulatory elements in promoter regions of *TaPSTOL1* orthologs. The color patterns indicate the distribution of different cis-elements across the orthologs. Legend on the left side shows the number of cis-elements and the respective colors on the map.

Differences in gene regulation and function can be understood by identifying cis-elements and their distribution patterns in the promoter region. After identifying cis-elements in the 1500 bp upstream promoter regions of the wheat *PSTOL1* orthologs, it was found that 24 cis-regulatory elements were involved in biotic and abiotic stress, 7 in growth and development, while 10 were associated with phytohormone response (Figure 3). G-Box cis-element is involved in the biotic and abiotic responses, while the ABRE element is involved in phytohormone response. Both of these cis-elements are distributed in almost all orthologs. G-Box prevailed in maximum number in the TraesCS3D02G014600 gene. After that, both G-Box and ABRE elements are abundant in the TraesCS3D02G261800 gene.

Most orthologs present phytohormone response-producing elements (CGTCA-motif, TCACG-motif) and growth and development responsive CAT-box. Contrary to this, the following other elements show a lower distribution among all orthologs: biotic and abiotic responsive elements (ATCT-motif, I-box, chs-CMA2a, GA-motif, ATC-motif, Box II, CAG-motif, GTGGC, and LAMP-element), growth and development responsive elements (circadian, HD-Zip 1, MSA-like, and RY-element), and the phytohormone response generating elements TGA-box.

### 3.4. Evolutionary Analysis of Wheat PSTOL1 Orthologs with Ancestral Species

**Figure 4:**
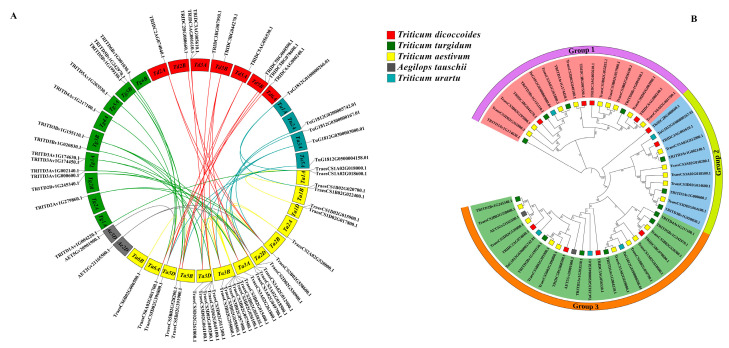
Syntenic and evolutionary analysis of *TaPSTOL1* orthologs with ancestral species. (**A**) Synteny analysis. (**B**) Phylogenetic tree of 22 *Triticum aestivum* L. *PSTOL1* orthologs with *Aegilops tauschii*, *Triticum urartu*, *Triticum dicoccoides*, and *Triticum turgidum*. Red-colored rectangles represent *T. dicoccoides*, green for *T. turgidum*, gray for *A. tauschii*, blue for *T. urartu*, and yellow for *T. aestivum* L.

Hexaploid wheat contains more than 85% of repetitive DNA due to two major polyploidization events that occurred thousands of years ago. Studying the duplication events and syntenic blocks among hexaploid wheat and its ancestors can help to better understand the gain in the function of *T. aestivum* and the evolutionary relationship with its diploid and tetraploid ancestors. Analysis of 22 *TaPSTOL1* orthologs with ancestors in synteny identified 80 gene pairs (Figure 4A). According to the study of [52], only the first transcripts of the duplicates were retained, while other splice variants were eliminated. After removing the 34 self-syntenic gene pairs, the phylogenetic tree was made on MEGAX. The phylogenetic tree classified *PSTOL1* genes into three groups (Figure 4B).

According to these analyses, 3 gene pairs were found among *Aegilops tauschii* and *Triticum aestivum*, 14 between *Triticum dicoccoides* and *Triticum aestivum*, 19 between *Triticum turgidum* and *Triticum aestivum*, and 10 in *Triticum urartu* and *T. aestivum*. This emphasizes that wheat *PSTOL1* genes were retained more from *T. turgidum*, *T. dicoccoides*, *T. urartu*, and *A. tauschii*, respectively. A and B sub-genomes of hexaploid wheat had fewer *PSTOL1* genes than wheat progenitors. This indicates that some *PSTOL1* genes might have been lost during polyploidization. At the same time, the D sub-genome showed an increased gene number in hexaploid wheat. This indicates that copy number increases during wheat polyploidization events. To further understand the evolutionary relationship and selection pressure on the genes, nonsynonymous and synonymous substitution rates (Ka/Ks) were calculated for the identified duplicates among wheat ancestors. Ka/Ks < 1 indicates purifying or negative selection. Ka/Ks = 1 implies neutral selection, while Ka/Ks > 1 suggests positive or natural selection. All gene pairs had Ka/Ks < 1, meaning that these genes underwent strong negative or purifying selection (Supplementary Table S1). With Ka/Ks, the divergence time of these genes was calculated between 0.5 and 208 million years ago.

### 3.5. Expression Patterns of Wheat PSTOL1 Orthologs in Transcriptomic Data

**Figure 5:**
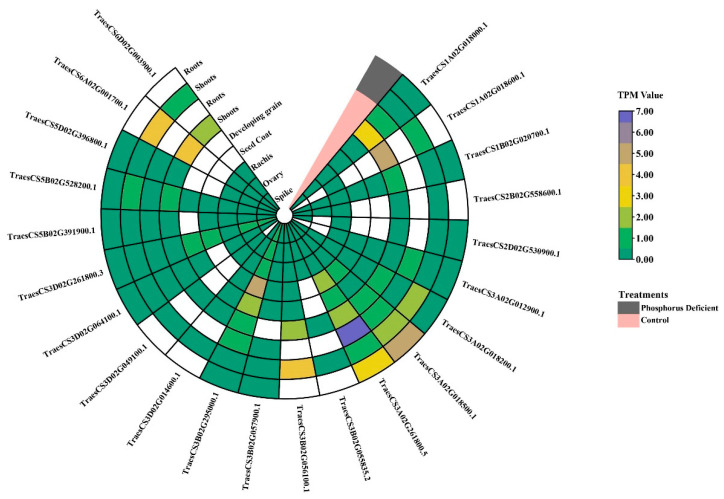
Gene expression profiling of *TaPSTOL1* in root and shoot in phosphorus-deficient treatment, and root, shoot with other tissues under control treatment. Color patterns illustrate the transcriptome abundance of these genes in the form of TPM values.

To understand the response to phosphorus starvation of *PSTOL1* in wheat, we investigated the expression pattern of *TaPSTOL1* from the root and shoot tissues under different treatments by analyzing the transcriptome data (Figure 5). Heatmap of RNA-seq data evidenced that most of *TaPSTOL1* were differentially expressed in vegetative tissues (root and shoot). Both A and B genomes *TaPSTOL1* showed a higher expression pattern, which suggests the A and B genomes may additively contribute to *TaPSTOL1’s* functional role under phosphorus deficit conditions. Whereas some *TaPSTOL1* demonstrated higher expression in root than shoot tissue, e.g., TraesCS3A02G261800 and TraesCS3A02G018500, we also noticed the reciprocal role of a few genes, e.g., TraesCS3B02G056100 was highly expressed in shoot in comparison to root tissues. These results reveal the dynamic role of some *TaPSTOL1* genes in shoot and root phosphorus uptake. Moreover, TraesCS3A02G018500 showed high expression in both root and shoot tissues under phosphorus starvation conditions, as compared to control shoot and root.

Upregulation and down-regulation of *TaPSTOL1* gene expression in vegetative tissues under both growth conditions (control and P starvation) were observed. For example, TraesCS3A02G261800 and TraesCS3B02G295000 genes were downregulated in both roots and shoots under phosphorus deficit. Contrary to this, TraesCS3A02G018500 showed upregulation in both root and shoot tissues under phosphorus starvation conditions. These results indicate the differential behavior (expressed or repressed) of wheat *PSTOL1* genes in phosphorus deficit response. All 22 *TaPSTOL1* genes showed minimal expression in rachis, ovary, spike, and developing grain while showing somewhat higher expression in the seed coat (Figure 5).

### 3.6. Validation of Expression Levels of Wheat PSTOL1 Genes under Different Phosphorus Regimes by Quantitative PCR

**Figure 6:**
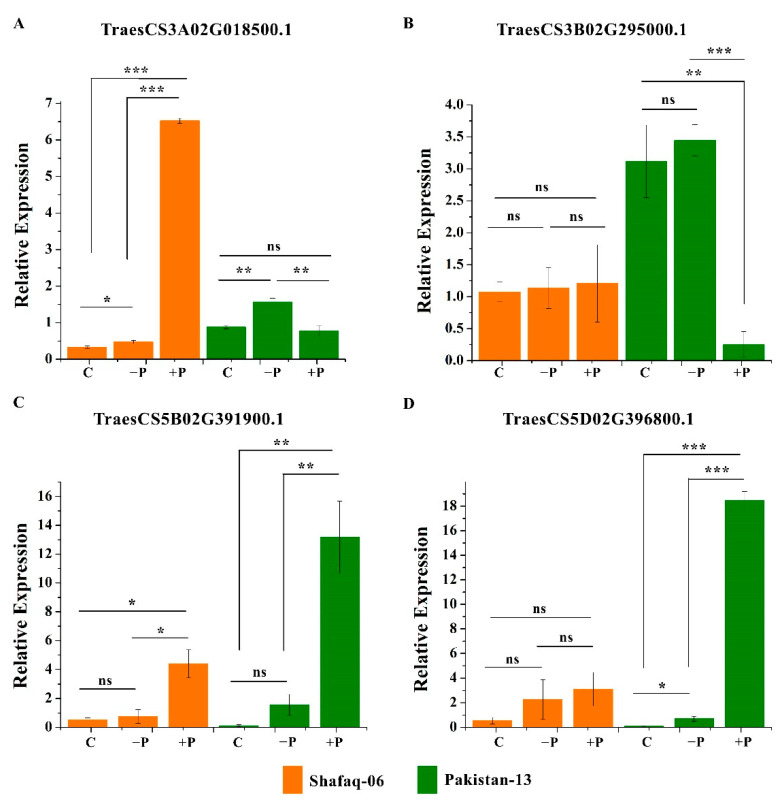
Relative expression of four genes under three different phosphorus treatments. Three biological replicates were used. *Actin* (reference gene) was used to normalize the data. Asterisks on bars indicate significant difference determined by *t*-test with *p* < 0.05 (*), *p* < 0.01 (**), and *p* < 0.001 (***).

We further validated the expression pattern of *TaPSTOL1* genes under different phosphorus regimes (Figure 6). Based on transcriptome data, four genes highly differentially expressed, i.e., TraesCS3A02G018500, TraesCS3B02G295000, TraesCS5B02G391900 and TraesCS5D02G396800 were selected for qPCR validation. These genes revealed dynamic expression patterns under different phosphorus regimes. Among the four *TaPSTOL1* genes in Pakistan-13, TraesCS3B02G295000 showed down-regulation under phosphorus-sufficient condition as compared to control plants, while TraesCS3A02G018500 did not show any difference as compared to control plants (Figure 6A,B). TraesCS5B02G391900 and TraesCS5D02G396800 demonstrated the highest expression levels under phosphorus-sufficient conditions as compared to control (Figure 6C,D). In Pakistan-13, TraesCS3A02G018500 and TraesCS5B02G391900 showed an increased expression compared to control plants under phosphorus-deficient conditions, whereas, TraesCS3B02G295000 and TraesCS5D02G396800 did not show any difference as compared to control plants under phosphorus-deficient conditions. The expression level of these genes in the examined tissues showed a consistent pattern with the transcriptome data.

### 3.7. Genotype-Dependent Gene Expression of Wheat PSTOL1 Genes under Different Phosphorus Regimes

We also examined the expression pattern of TraesCS3A02G018500, TraesCS3B02G295000, TraesCS5B02G391900 and TraesCS5D02G396800 in another wheat cultivar, i.e., Shafaq-06 (Figure 6). TraesCS3A02G018500, TraesCS5B02G391900, and TraesCS5D02G396800 showed an increase in their expression compared to control plants under phosphorus-sufficient conditions, whereas the expression of these genes did not change as compared to control plants in phosphorus-deficient conditions. However, TraesCS3B02G295000 did not show any difference as compared to control in both phosphorus regimes. Interestingly, we noticed that both wheat cultivars, i.e., Pakistan-13 and Shafaq-06, showed differences in the expression of the tested genes. In Shafaq-06, TraesCS3A02G018500 showed a very strong increase in the expression under phosphorus-sufficient conditions as compared to control, whereas, TraesCS3A02G018500 showed an increase in phosphorus-deficient conditions in Pakistan-13. Furthermore, TraesCS3B02G295000 showed a very strong decrease in the expression under phosphorus-sufficient condition in Pakistan 13 but did not change in Shafaq-06. It is also important to notice that basal TraesCS3B02G295000 expression was higher in Paksitan-13 as compared to Shafaq-06. TraesCS5B02G391900 and TraesCS5D02G396800 showed similar patterns of gene expression in Pakistan-13 and Shafaq-06 under different phosphorus regimes, but their expression levels were higher in Pakistan-13 under phosphorus-sufficient conditions as compared to Shafaq-06. These results indicate the specific regulation of *TaPSTOL1* genes in different phosphorus regimes, and their regulation might depend on the genetic makeup of the wheat cultivars.

### 3.8. Co-Localization of TaPSTOL1 Orthologs with Phosphorus Deficiency Responsive QTLs

**Figure 7:**
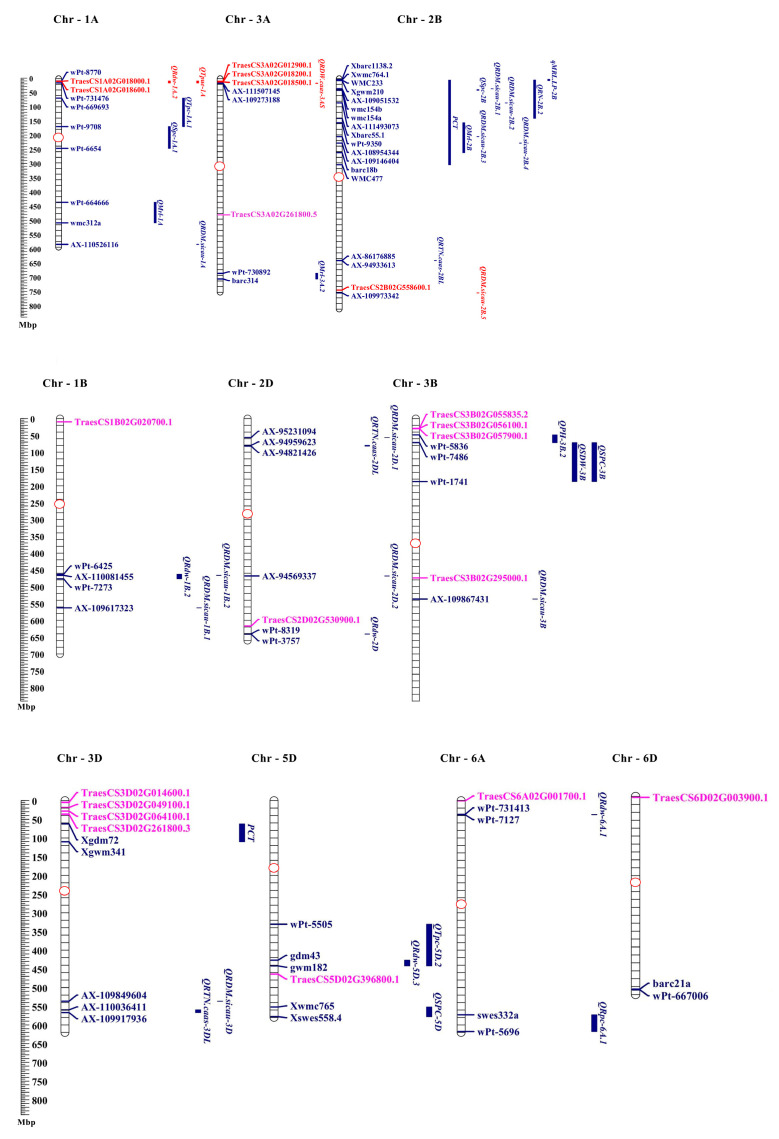
Co-localization of six *TaPSTOL1* orthologs on three chromosomes, i.e., 1A, 3A, and 2B. Scale on the left-hand side shows the physical position of these genes in Mbp. Genes located within the QTLs are illustrated with red color, while genes and QTLs not linked are represented with pink and blue colors. Red circle represents the centromere position.

To further understand and validate the function of *TaPSTOL1* genes, these orthologs were plotted against the QTLs related to phosphorus deficiency. These QTLs were identified for their involvement in maximum root length (MRL), root dry weight (RDW), shoot, root, and total phosphorus content (SPC, RPC, TPC), root diameter (RDM), root tip number (RTN), number of axial root length (RN) under phosphorus-deficient conditions. All 22 genes were mapped against QTLs related to the phosphorus regime on 10 different chromosomes, i.e., 1A, 1B, 2B, 3A, 3B, 3D, 5B, 5D, 6A, and 6D (Figure 7, and Supplementary Figure S1). However, only six genes were found to be co-localized with the reported QTLs. Five genes were on chromosomes 1A and 3A, while one gene was on chromosome 2B. TraesCS1A02G018000 and TraesCS1A02G018600 were located within QRdw-1A.2. TraesCS3A02G012900, TraesCS3A02G018200, TraesCS3A02G018500 were in close proximity of QRDW.caas-3AS. In the B sub-genome, TraesCS2B02G558600 was linked with QRDM.sicau-2B.5 (Figure 7).

### 3.9. Morpho-Physiological Evaluation and Phosphorus Uptake of Wheat Cultivars under Different Phosphorus Regimes

**Figure 8:**
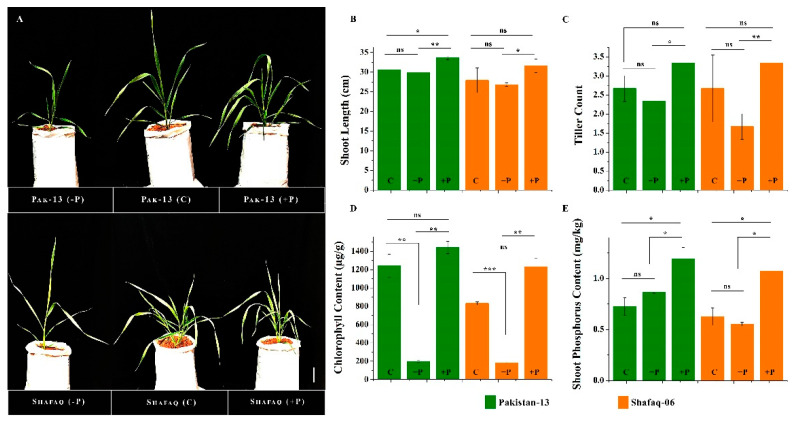
Plants’ response toward P-sufficient (+P), control (C) and P-starvation (−P) conditions. (**A**) Pakistan-13 and Shafaq-06 under low phosphorus (−P), control (C) and high phosphorus (+P). Bar = 5 cm. (**B**) For both (Pakistan-13 and Shafaq-06) genotypes shoot length (cm), (**C**) tillers count, (**D**) chlorophyll contents, and (**E**) shoot phosphorus contents. Presented data are the mean of five biological replicates. Asterisks on bars indicate significant difference determined by t-test with *p* < 0.05 (*), *p* < 0.01 (**), and *p* < 0.001 (***).

Pakistan-13 and Shafaq-06 showed differences in the expression of selected *TaPSTOL1* genes. We examined the morpho-physiological traits, i.e., shoot length (cm), tiller count, chlorophyll content, and shoot phosphorus content, under different phosphorus regimes in both wheat cultivars (Figure 8). Both cultivars revealed a similar trend for shoot length and tiller number under +P, −P, and C treatments. Plants under phosphorus-sufficient condition demonstrated better plant growth in shoot length and tiller count than control and phosphorus-deficient condition (Figure 8A–C). Shoot length and tiller count showed significant differences between +P and −P conditions for both cultivars. Further, significant performance was observed in Pakistan-13 for shoot length compared to Shafaq-06 under −P condition. Chlorophyll content was detected relatively low in −P condition for both cultivars, whereas Pakistan-13 showed slightly higher levels of chlorophyll content than Shafaq-06 (Figure 8D). We further quantified the phosphorus contents in the shoots of both cultivars (Figure 8E). Both Pakistan 13 and Shafaq-06 showed an increase in phosphorus in their shoots under phosphorus-sufficient conditions as compared to control. However, it appears that Pakistan 13 accumulated slightly more phosphorus in the shoot as compared to Shafaq-06. Furthermore, under phosphorus-deficient conditions, it also appears that phosphorus contents are higher in Pakistan 13 as compared to Shafaq-06. These results suggest that the Pakistan-13 is efficient for P uptake under phosphorus starvation and performs better than Shafaq-06.

### 3.10. High-Throughput Root Phenotyping of Wheat Cultivars under Different Phosphorus Treatments

**Figure 9:**
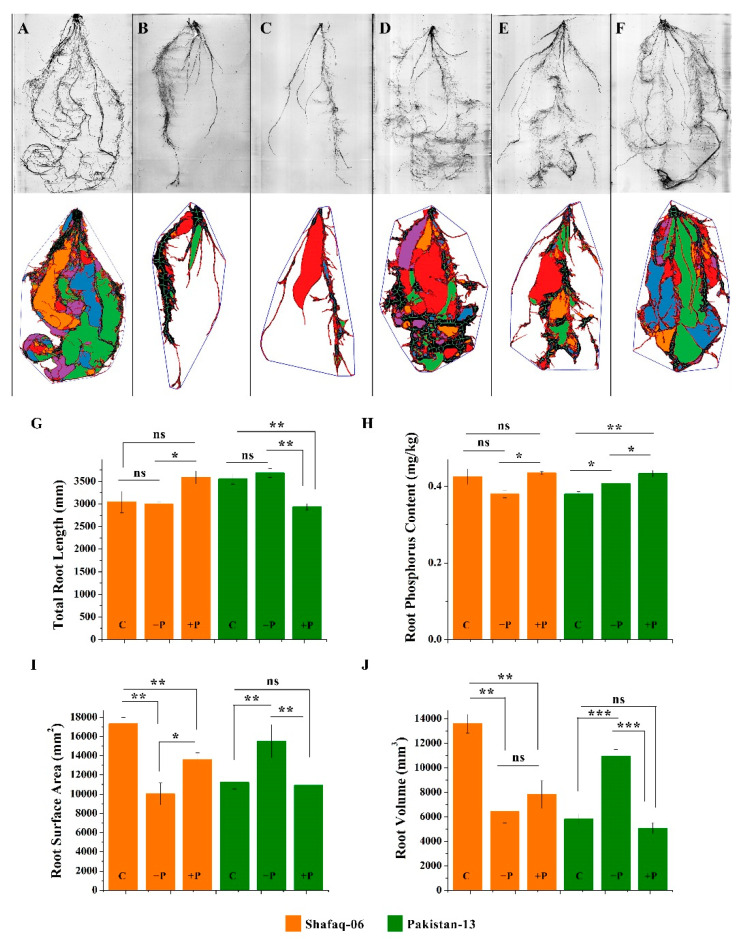
High-throughput root phenotyping. (**A**–**C**) Shafaq-06 scanned roots and RhizoVision Explorer roots under three different phosphorus growing conditions. (**D**–**F**) Pakistan-13 scanned roots and RhizoVision Explorer roots under three different phosphorus growing conditions. (**G**) Total root length (cm). (**H**) Root surface area (mm^2^). (**I**) Root phosphorus content (mg/kg). (**J**) Root volume (mm^3^). Asterisks on bars indicate the significant differences determined by *t*-test with *p* < 0.05 (*), *p* < 0.01 (**), and *p* < 0.001 (***).

Because Paksitan-13 and Shafaq-06 showed differences in the *TaPSTOL1s* expression and the morpho-physiological traits, we further investigated the effect of different phosphorus regimes on the root morphology in both cultivars (Figure 9). High-throughput root phenotyping was performed using scanned root images and analyzing those images by RhizoVision Explorer. The total root length, root surface area, root volume, and phosphorus content were calculated (Figure 9). Pakistan-13 and Shafaq-06 showed differences in root morphology under control conditions. Under the phosphorus-deficient condition, Pakistan-13 revealed prominent root architecture, i.e., root length, root surface area, and root volume, as compared to Shafaq-06 (Figure 9A–F). Pakistan-13 had elongated roots, higher root surface area, and root volume in -P treatment, while Shafaq-06 exhibited stunted root growth, lower root surface area, and volume (Figure 9G,I,J). Pakistan-13 roots also revealed a higher P uptake than Shafaq-06 under phosphorus starvation treatment (Figure 9H). However, phosphorus content is comparable between Pakistan-13 and Shafaq-06 under phosphorus-sufficient treatment (Figure 9H). Despite the similar levels of phosphorus in the root of Pakistan 13 and Shafaq-06 under phosphorus-sufficient conditions, Pakistan 13 and Shafaq-06 differ in the total root length, root surface area, and root volume. These results suggest the activation of different genetic mechanisms in response to different phosphorus regimes between Pakistan-13 and Shafaq-06.

## 4. Discussion

Phosphorus deficiency is one of the factors that excessively increase farmers’ costs because it must be applied as fertilizers, which is not cost-effective [53]. Plants are trying to survive under phosphorus deficiency by increasing phosphorus uptake, which can be achieved by modifying their root system architecture, changing rhizospheres, interacting with microorganisms, or working around with the transport of internal phosphorus and mobilization [53]. Over time, multiple genes have been identified in different plants that can influence plants’ responses to many of such macronutrient deficiencies. With all the tools and genomes at our disposal, identifying genetic variants of many genes, even in very complex genomes such as wheat, has become more accessible. In this study, we have identified 22 *PSTOL1* orthologs in the wheat genome and highlighted their potential role in wheat morpho-physiological traits and root architecture.

*PSTOL1* gene belongs to the receptor-like kinases (RLK family) [54]. *OsPSTOL1* gene was first identified upon sequencing of *PuP1* QTL in Kasalath and Nipponbare rice varieties; *OsPSTOL1* is responsible for early root growth in phosphorus-deficient soil and increases grain yield exponentially [16]. This study identified 22 orthologs of *PSTOL1* in *Triticum aestivum* L. through BLAST search, conserved motif, and domain analysis. Previously, six orthologs were identified in *Sorghum bicolor* [1], and six were identified in *Zea mays* [53], a significantly lower number compared to wheat orthologs. This indicates that the whole-genome duplication events and natural purifying selection lead to a higher diversity of *PSTOL1* genes in wheat, and *PSTOL1* genes in wheat potentially have diverse functions compared to other plant species. Based on phylogenetic analysis with other crop species, *TaPSTOL1* genes indicated closed evolutionary relationships with *B. distachyon*, *S. bicolor*, and *A. thaliana* while diverging relations with *O. sativa* and *Z. mays*. This evolutionary relationship revealed that *PSTOL1* might play a similar role in plant development and phosphorus uptake genetic mechanism among these species. Contrary to this study, Milner et al. [55] reported that the wheat genome has only one *PSTOL1* homolog on chromosome 5A, but it is highly conserved in different wheat accessions. They also showed that *OsPSTOLI* homolog in wheat (*TaPSTOL1*) is regulated under phosphorus limiting conditions, and *TaPSTOL1* is involved in root growth, flowering time, and seed yield [55]. It is important to mention here that Milner et al. used the wheat reference sequence of IWGSC (2014) for BLAST and orthologs identification. As genome assembly of *Triticum aestivum* L. is known to be continuously updated due to its vast size; hence the gene on the 5A chromosome said to be *TaPSTOL1* by the previous study can be seen as a low confidence gene according to the new reference genome IWGSC 1.1 (2017). Consequently, we identified 22 orthologs of *PSTOL1* in wheat using the IWGSC 1.1 (2017).

Domain analysis revealed that 22 *TaPSTOL1* orthologs contained the conserved Ser/Thr kinase domain, as shown in other crop species, i.e., sorghum and maize [1,53]. Along with this conserved domain, similar to *OsPSTOL1* and *SbPSTOL1,* wheat *PSTOL1* orthologs also included other domain features. One was the presence of wall-associated kinases or WAKs domain, which links the cytoplasm and the pectin fraction of the cell wall and is also responsible for generating stress responses [56,57]. WAKs are also found to cause root extension and elongation in barley (*Hordeum vulgare*), along with the interaction with other genes as a stress response for alteration of root development [58]. According to Hafnagel et al. [1], the interaction of the cell wall and cell membrane may cause increased root surface area and phosphorus uptake during phosphorus deficit conditions. Our study also highlighted the diverse role of the *TaPSTOL1* genes in phosphorus use efficiency and root architecture under phosphorus deficiency. Furthermore, this study also demonstrated the differential expression of *TaPSTOL1* genes upon phosphorus starvation treatment (Figure 5 and Figure 6). We also observed higher expression of *TaPSTOL1* orthologs in a phosphorus efficient cultivar (Pakistan-13) compared to an inefficient cultivar (Shafaq-06), showing the genetic diversity of wheat germplasm in phosphorus use efficiency. Genetic and epigenetic variations in wheat cultivars could lead to differences in root morphology and stress response [59]. Indeed, we do see the genetic diversity between Pakistan 13 and Shafaq-06 for the phosphorus use efficiency and root morphology (Figure 8 and Figure 9). Furthermore, qRT-PCR results showed that few *TaPSTOL1* genes were upregulated, while few were downregulated in phosphorus efficient variety (Pakistan 13) under −P treatment (Figure 6). This specific and differential regulation of wheat *PSTOL1* genes under different phosphorus regimes suggests the important role of *TaPSTOL1* genes in acclimatization to variable soil nutrient profiling. This provides the evidence of multi-functional roles of *PSTOL1* genes under specific stimuli in wheat.

Moreover, meta-QTL analysis was performed to understand the multiple functional involvements of *PSTOL1* genes in wheat. Two genes (*TraesCS1A02G018000* and *TraesCS1A02G018600*) on chromosome 1A, three genes (*Traes3A02G012900*, *Traes3A02G018200, Traes3A02G018500*) on 3A and one gene (*TraesCS2B02G558600*) on 2B, were co-localized with the phosphorus related QTLs (Figure 7). These co-localized QTLs related to phosphorus starvation indicate multiple roles of *TaPSTOL1s* for different phenotypes. In addition, co-localized QTLs identified on the A and B genome of wheat support our previous conclusion that both A and B genomes may additively contribute to the *PSTOL1* functional role in wheat under phosphorus deficit conditions. Interestingly, *TraesCS3A02G018500* was co-localized with the QTL of dry root weight. Quantitative PCR results showed the upregulation of *TraesCS3A02G018500* in Pakistan-13 (P-efficient cultivar) under −P treatment. Morpho-physiological phenotypic evaluation of Pakistan-13 demonstrated longer shoot and root length, higher chlorophyll content, abundant accumulation of P in root and shoot tissues under phosphorus-deficient conditions. Together, we propose the multi-functional role of the *TraesCS3A02G018500* gene in wheat growth, maintaining better physiology, and efficiently uptake phosphorus in response to phosphorus starvation stimuli.

In brief, our study highlighted the potential role of *TaPSTOL1* genes in phosphorus use efficiency under a P-deficit environment. Further investigations are required to systematically characterize the function of all *PSTOL1* genes in wheat phosphorus use efficiency.

## 5. Conclusions

In the present study, we identified and characterized 22 TaPSTOL1 gene family members using phylogenetic, motif, and domain analysis. The higher expression level in the root tissues suggested that *TaPSTOL1* genes played an important role in phosphorus use efficiency. Pakistan-13 showed increased expression of *TaPSTOL1* genes and more profound root growth and improved architecture with increased phosphorus uptake under phosphorus starvation. Conclusively, this study highlighted the potential role of *TaPSTOL1* genes. These genes might control the morphology (i.e., root architecture) and physiology (i.e., chlorophyll content, root and shoot P content) of phosphorus efficient cultivar (Pakistan-13) under phosphorus starvation conditions, which might help the cultivar to adapt under worse environmental conditions. These results also indicate the genetic diversity for morpho-physiological traits (especially root architecture and phosphorus acquisition) of both cultivars. This study motivates the investigation of the biological and cellular functions of each *PSTOL1* gene in hexaploid wheat, which could ultimately lead to a long-term adaptation and sustainable production of wheat under low P-treatment. Utilization and selection based on the *PSTOL1* gene could improve the phosphorus use efficiency in future wheat varieties.

## Data Availability

Not applicable.

## Supplementary Materials

The following supporting information can be downloaded at: https://www.mdpi.com/article/10.3390/genes13030487/s1, Table S1: Ka/Ks analysis and divergence time (MYA) of *TaPSTOL1* genes with wheat ancestors; Table S2: List of primers for expression profiling; Table S3: Protein features of *TaPSTOL1* genes; Table S4: Conserved motif sequences; Figure S1: Chromosomal location and segmental duplicates.
