# Supplementary material for: Role of Wheat Phosphorus Starvation Tolerance 1 Genes in Phosphorus Acquisition and Root Architecture
Source: Genes (Basel). 2022 Mar 10;13(3):487. doi: 10.3390/genes13030487 (PMC8950872; doi:10.3390/genes13030487)

Research Article

Distinct Role of Wheat *Phosphorus Starvation Tolerance 1* (*TaPSTOL1*) Gene in Phosphorus Uptake and Root Architecture

Hina Abbas [1,†], Muhammad Kashif Naeem [1,†], Marya Rubab [1,2], Emilie Widemann [3], Muhammad Uzair [1], Nageen Zahra [1], Bilal Saleem [1], Amna Abdul Rahim [1], Safeena Inam [1], Muhammad Imran [4], Farhan Hafeez [5], Muhammad Ramzan Khan [1,*] and Sarfraz Shafiq [5,6,*]

1 National Institute for Genomics and Advanced Biotechnology, National Agricultural Research Centre, Islamabad, 44000, Pakistan

2 Department of Biotechnology, University of Kotli, Azad Jammu and Kashmir, 11100, Pakistan

3 Institut de Biologie Moléculaire des Plantes, CNRS-Université de Strasbourg, 67084 Strasbourg, France

4 Department of Environmental Sciences, Abbottabad Campus, COMSATS University Islamabad, University Road, Abbottabad 22060, Pakistan

5 Department of Anatomy and Cell Biology, University of Western Ontario, 1151 Richmond St., London, ON N6A5B8, Canada

† equal contribution

*Correspondence: mrkhan@parc.gov.pk, sshafiq2@uwo.ca

Table S1: Ka/Ks analysis and divergence time (MYA) of Ta*PSTOL1* genes with wheat ancestors.

Genes	Genes	Ka/Ks	Divergence Time (MYA)
AET2Gv21165500	TraesCS2B02G558600	0.330	8.57
AET2Gv21165500	TraesCS2D02G530900	0.732	1.66
AET5Gv20901900	TraesCS5D02G396800	0.676	4.10
TRIDC2AG074040	TraesCS2B02G558600	0.293	12.63
TRIDC2AG074040	TraesCS2D02G530900	0.267	13.53
TRIDC2BG080660	TraesCS2B02G558600	0.350	5.41
TRIDC3AG001610	TraesCS3A02G012900	0.630	0.58
TRIDC3AG005610	TraesCS3D02G049100	0.192	15.79
TRIDC3BG044270	TraesCS3A02G261800	0.071	9.83
TRIDC3BG044270	TraesCS3B02G295000	0.097	1.20
TRIDC3BG044270	TraesCS3D02G261800	0.156	8.98
TRIDC3BG007590	TraesCS3D02G049100	0.185	5.50
TRIDC5AG056530	TraesCS5B02G391900	0.263	10.17
TRIDC5AG056530	TraesCS5D02G396800	0.168	9.67
TRIDC5BG078600	TraesCS5B02G528200	0.615	6.96
TRIDC5BG060590	TraesCS5B02G391900	0.615	0.87
TRIDC5BG060590	TraesCS5D02G396800	0.228	10.78
TRIDC6AG000240	TraesCS6A02G001700	0.307	0.52
TRITD1Av1G004220	TraesCS1A02G018600	0.240	21.65
TRITD2Bv1G245340	TraesCS2D02G530900	0.349	7.64
TRITD3Av1G000600	TraesCS3A02G018500	0.119	24.28
TRITD3Av1G174450	TraesCS3A02G261800	0.744	5.81
TRITD3Av1G002140	TraesCS3A02G012900	0.181	9.07
TRITD3Av1G174630	TraesCS3D02G261800	0.263	208.63
TRITD3Bv1G155110	TraesCS3A02G261800	0.295	11.27
TRITD3Bv1G155110	TraesCS3B02G295000	0.640	26.51
TRITD3Bv1G155110	TraesCS3D02G261800	0.544	36.44
TRITD3Bv1G020830	TraesCS3D02G064100	0.526	1.75
TRITD4Av1G217100	TraesCS5B02G528200	0.403	14.28
TRITD5Bv1G199150	TraesCS5B02G391900	0.583	1.50
TRITD5Bv1G199150	TraesCS5D02G396800	0.224	10.70
TRITD6Bv1G001030	TraesCS6A02G001700	0.263	8.37
TraesCS1A02G018000	TuG1812G0100000266	0.381	14.71
TraesCS1B02G020700	TuG1812G0100000266	0.127	14.10

Table S2: List of primers for expression profiling

S. No.	Gene	Primer Name	Primer Seq.
1	TraesCS3A02G018500	P3A500-F	TCTGAAGGAATGAGACGGGC
	TraesCS3A02G018500	P3A500-R	TTGGGTGCTAGGAGTTGTCG
2	TraesCS3A02G261800	P3A800-F	TCTCTTGATATGCCACCGGC
	TraesCS3A02G261800	P3A800-R	TGATTCAGGCACATGGAGGG
3	TraesCS3B02G295000	P3B5K-F	CCAGCACCTCCACTTCAACT
	TraesCS3B02G295000	P3B5K-R	TGAGGCGTTCTTCTTGCAGT
4	TraesCS5B02G391900	P5B900-F	ATCTCAGATGCTGCCATCCC
	TraesCS5B02G391900	P5B900-R	GCTCCAGTCAGCTGCTATGT
5	TraesCS5D02G396800	P5D800-F	TTCCCTCTCCAGCTCCTTGA
	TraesCS5D02G396800	P5D800-R	TGATGGTGTCTTGGCCTGAG

Table S3: Protein Features of *TaPSTOL1* genes.

Genes	Transcripts	Chr No	Start Position (bp)	End Position (bp)	Gene Size (bp)	Strand	Protein (AA)	Isoelectric Point	Molecular Weight (Kda)
TraesCS1A02G018000	TraesCS1A02G018000.1	1A	9,359,231	9,363,721	1905	+	634	7.79	70802.77
TraesCS1A02G018600	TraesCS1A02G018600.1	1A	9,553,967	9,556,916	1938	+	645	7.13	72380.39
TraesCS1B02G020700	TraesCS1B02G020700.1	1B	9,592,944	9,596,685	1415	-	437	8.25	49406.25
TraesCS2B02G558600	TraesCS2B02G558600.1	2B	752,831,383	752,834,890	1932	+	643	8.24	70888.45
TraesCS2D02G530900	TraesCS2D02G530900.1	2D	617,074,366	617,078,126	2143	+	632	7.46	69917.22
TraesCS3A02G012900	TraesCS3A02G012900.1	3A	9,610,472	9,613,044	1998	-	625	8.03	69611.1
TraesCS3A02G018200	TraesCS3A02G018200.1	3A	11,412,724	11,415,681	1980	+	418	9.14	46812.23
TraesCS3A02G018500	TraesCS3A02G018500.1	3A	11,471,664	11,472,966	1146	+	381	8.83	43148.18
TraesCS3A02G261800	TraesCS3A02G261800.5	3A	484,637,356	484,654,579	1938	-	645	6.84	71516.53
TraesCS3B02G055835	TraesCS3B02G055835.2	3B	29,521,586	29,530,365	1815	-	604	8.4	68176.66
TraesCS3B02G056100	TraesCS3B02G056100.1	3B	29,614,087	29,629,692	1929	+	642	8.56	71009.86
TraesCS3B02G057900	TraesCS3B02G057900.1	3B	30,198,134	30,208,607	1938	+	645	8.94	72270.54
TraesCS3B02G295000	TraesCS3B02G295000.1	3B	473,694,773	473,709,342	1824	-	607	7.15	67019.24
TraesCS3D02G014600	TraesCS3D02G014600.1	3D	5,013,282	5,016,625	2603	+	620	8.17	69314.66
TraesCS3D02G049100	TraesCS3D02G049100.1	3D	18,815,473	18,847,345	1950	+	649	8.21	72083.98
TraesCS3D02G064100	TraesCS3D02G064100.1	3D	28,326,340	28,329,337	1950	-	625	8.31	70586.88
TraesCS3D02G261800	TraesCS3D02G261800.3	3D	363,598,032	363,608,944	1980	-	659	5.81	73235.33
TraesCS5B02G391900	TraesCS5B02G391900.1	5B	570,930,921	570,934,404	2364	-	720	8.31	79132.44
TraesCS5B02G528200	TraesCS5B02G528200.1	5B	687,160,010	687,163,240	2243	-	614	8.38	68819.42
TraesCS5D02G396800	TraesCS5D02G396800.1	5D	464,073,741	464,076,774	1986	-	661	7.77	72671.16
TraesCS6A02G001700	TraesCS6A02G001700.1	6A	811,604	816,032	1902	+	633	8.45	69474.17
TraesCS6D02G003900	TraesCS6D02G003900.1	6D	2,047,133	2,051,430	1866	-	621	7.23	68365.78

Table S4: Conserved Motif Sequences

Motifs	Sequence
Motif 1	DIALGIARGMEYLHQGCNQRILHFDIKPHNILLDYNFNPKISDFGLAKLC
Motif 2	ZSIITLTAARGTMGYIAPELYSRNFGGVSYKSDVYSFGMLVLEMVSGRRN
Motif 3	EGEEFINEVATIGRIHHVNIVRLLGFCSEGSRRALIYEFMPNGSLEKYIF
Motif 4	EKARKLAIVALWCIQWNPRNRPSMTKVVNMLTGSLQBLQMPPKPFVSSEN
Motif 5	RYNEEIHLKVEMFLKTYGTSKPTRYTFSEVKKITRRFKDKLGQGGFGSVY
Motif 6	DPSVESQNEVYLPEWIYEKVITGZELVLT
Motif 7	KGELPBGVPVAVKML
Motif 8	KIIAATSSVAAFVVLLLMVATALYLSLKT
Motif 9	NDGAYASAAWDDQDFFKNCPPSRCSKHGP
Motif 10	CQKCELNGQRCAFSSQRNZTFCLPQPHG
Motif 11	CSGQDTILLHPLLGPCNVTAIBYTGASLNITPLVEACTVIQ
Motif 12	PRELLGPEKLL
Motif 13	QYFPQWIYEHLDEYCISASEINGEITEVVRKMIVVGLWCIQLSSTDRPTM
Motif 14	PSAADSIAGPISCLSNTTHFSYLV
Motif 15	TSSPPADTGHHCAPHYSGAGKLVGCSREFTPSGITQYPVYD
Motif 16	ATDAFMYDLPLDCKVISDAAJPIFGTGYYGSASKHGVEGG
Motif 17	KRRSKKIMLIGVTSAAAALLFACIYVLIWHRKGKRLWFLLC
Motif 18	EIRFPFRLESSNTSS
Motif 19	EDSAHNMDVQSSSSTKSEEISLVNSKILQ
Motif 20	GEGCSPKRCGNLTISDPFWL

Figure S1: Chromosomal location and segmental duplicates. 3D, 5B, 5D, 6A, and 6D represent chromosome numbers. The ruler on the left side indicates chromosome size in Mbps. Red-colored gene labels show segmental duplicates, and the blue lines indicate a duplicate link.

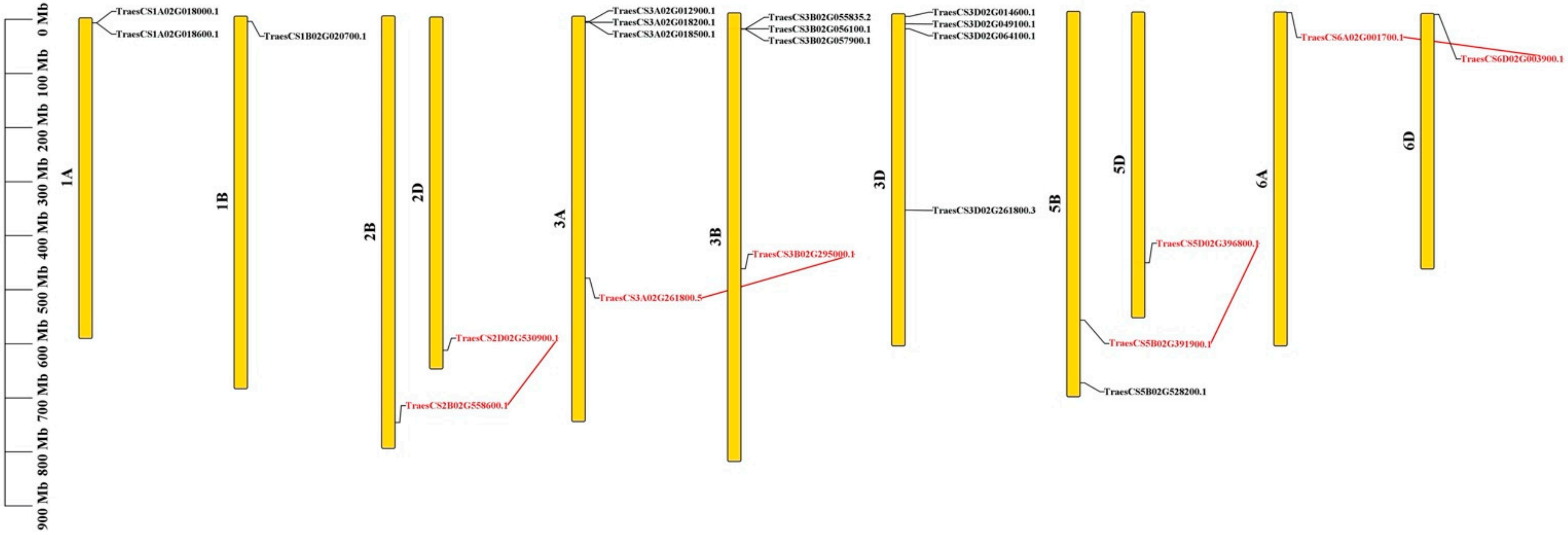

Supplement: Supplementary file 1 [file genes-13-00487-s001.zip › genes-1616065-supplementary.pdf]
